# Knowledge on birth control pills among married rural Indian women

**DOI:** 10.6026/9732063002001026

**Published:** 2024-09-30

**Authors:** Mahalakshmi B., Sithara Begum K., Aruna S., Ramani G., Malarkodi M., Anuradha M., Siva Subramanian N.

**Affiliations:** 1 Nootan College of Nursing, Sankalchand Patel University, Visnagar, Gujarat - 384315, India; 2 College of Nursing, Jazan University, Jazan, KSA; 3 Faculty of Nursing, SRMC & RI (DU), Porur, Chennai, Tamilnadu - 600116, India; 4 Kongunadu College of Nursing, Coimbatore, Tamilnadu - 641109; India; 5 KMCH College of Nursing, Coimbatore, Tamilnadu - 641048, India

**Keywords:** Birth control pills, contraceptive knowledge, rural women, socio-demographic factors

## Abstract

Despite the availability and benefits of birth control pills, rural areas in countries like India face challenges regarding contraceptive knowledge and utilization. Factors such as limited access to healthcare, cultural taboos, and educational barriers contribute to this issue. Addressing these gaps requires tailored reproductive health education and improved access to contraceptive services. This study aims to assess birth control pill knowledge among married women in rural areas of Mahesana District, Gujarat, India, considering socio-demographic factors and information sources. A descriptive research design was employed, with 100 married women sampled using convenience sampling. Data were collected through structured questionnaires covering socio-demographic variables and birth control pill knowledge. Descriptive and inferential statistics, including chi-square tests, were utilized for analysis. The mean knowledge score for birth control pills among surveyed women was 11.97 out of 20, corresponding to 59.85%. Significant associations were found between knowledge levels and age, number of children, religion, occupation, family-type, source of information, socioeconomic status and education level. Younger women and those with fewer children demonstrated higher knowledge levels. Additionally, women with higher education, access to healthcare, and diverse sources of information exhibited greater knowledge. The study reveals a substantial level of knowledge among rural married women regarding birth control pills, suggesting effective dissemination of contraceptive information in the studied region. Factors such as education, access to healthcare, and diverse information sources significantly influence contraceptive knowledge. These findings align with previous research, emphasizing the importance of tailored interventions and improved access to reproductive health services. Further research could explore additional factors influencing contraceptive knowledge and inform targeted interventions for improving reproductive health outcomes in rural areas.

## Background:

Despite the widespread availability and benefits of birth control pills, their optimal use depends heavily on individuals' knowledge and awareness. Unfortunately, in many rural areas of developing countries such as India, access to accurate information about contraception, including birth control pills, remains limited. This lack of knowledge can lead to misconceptions, myths, and barriers to the effective utilization of contraceptive methods, ultimately impacting women's reproductive health outcomes [1]. In rural regions of India, various factors contribute to the challenges surrounding contraceptive knowledge and utilization. These include limited access to healthcare facilities, cultural taboos surrounding discussions about reproductive health, socioeconomic disparities, and educational barriers [2]. Additionally, the patriarchal nature of society in some areas may restrict women's autonomy in making decisions about their reproductive health, further exacerbating the issue [3]. Addressing the gaps in knowledge and utilization of birth control pills among women in rural India requires multifaceted approaches. Comprehensive reproductive health education programs tailored to the needs of rural communities are essential for increasing awareness about contraception, debunking myths, and promoting informed decision-making [4]. Furthermore, ensuring the availability of contraceptive services and commodities at grassroots-level healthcare facilities is crucial for enhancing access and uptake among rural populations [5]. Therefore, it is of interest to assess the knowledge regarding birth control pills among married women residing in selected rural areas of Mahesana district, Gujarat, India.

## Methodology:

## Research design and type:

This study employed a descriptive research design [6, 7] to assess birth control pill knowledge among married women in rural areas of Mahesana District, Gujarat, India.

## Sample size and sampling:

A sample of 100 married women from rural areas of Mahesana District was selected using convenience sampling, ensuring accessibility and feasibility.

## Data collection:

Data were collected using a structured questionnaire administered face-to-face by trained researchers, covering socio-demographic variables and birth control pill knowledge (Table 1).

## Data analysis:

Descriptive statistics summarized socio-demographic characteristics and knowledge levels (Table 1), while inferential statistics, including chi-square tests, examined associations between demographic variables and knowledge levels. Analysis was conducted using SPSS.

## Results:

Table 2 shows, 83.4% women have knowledge regarding the Introduction of birth control pills, 52.6% women have knowledge regarding Mala N & Mala D, 63.5%women have knowledge regarding Ezy Pills, 22.5% women have knowledge regarding Saheli Pills, 67% women have knowledge regarding Side Effects of the birth control Pills, and 42% women have knowledge regarding Advantages & Disadvantages of birth control pills. Regarding over all the mean score for knowledge regarding birth control pills among the surveyed women was 11.97 out of a maximum score of 20, corresponding to a mean percentage of 59.85%. The standard deviation for the knowledge scores was 2.2717, indicating the variability of scores within the sample of 100 women.

The analysis of the data revealed significant associations between the knowledge level regarding birth control pills and various demographic variables among the surveyed women, as indicated by the chi-square values. Across different age groups, a notable association (χ^2^ = 9.5967, p < 0.05) was observed, with younger women demonstrating higher levels of knowledge compared to older age groups. Similarly, the number of children also exhibited a significant association (χ^2^ = 30.471, p < 0.05), with women having fewer children showing higher levels of knowledge. Furthermore, factors such as religion (χ^2^ = 9.7119, p < 0.05), occupation (χ^2^ = 9.7119, p < 0.05), type of family (χ^2^ = 10.548, p < 0.05), source of information (χ^2^ = 14.74, p < 0.05), socioeconomic status (χ^2^ = 5.777, p < 0.05), and education level (χ^2^ = 16.458, p < 0.05) were found to be associated with variations in knowledge levels among the surveyed women.

## Discussion:

Mahesana district, characterized by its predominantly rural landscape, provides a pertinent backdrop for this investigation. By delving into the socio-demographic profiles of the participants and their corresponding levels of knowledge, the study aims to shed light on factors influencing awareness and understanding of birth control methods in this context. Understanding the demographic composition of the study population, including age, educational background, occupation, and family structure, is fundamental for contextualizing the findings. These factors often play a pivotal role in shaping individuals' access to information and their attitudes towards contraception. Moreover, exploring the sources from which women acquire knowledge about birth control pills is essential for discerning the efficacy of existing dissemination channels and identifying potential avenues for outreach and education.

**Figure 1:**
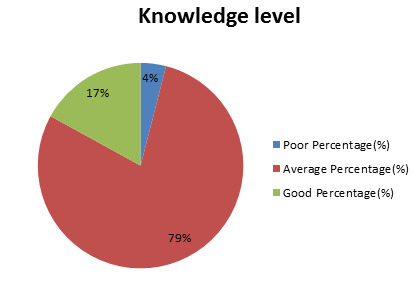
Knowledge Level of women regarding birth control pills.

We investigated the knowledge levels concerning birth control pills among married women in rural areas of Mahesana District, Gujarat, India. Our findings revealed that a significant percentage of participants possessed adequate knowledge about contraception. Specifically, 79% of the surveyed women demonstrated a thorough average understanding of birth control methods, including birth control pills (Figure 1). This observation is consistent with previous research conducted by Gupta *et al.* (2015) [8] and Alameer *et al.* (2022) [9]. This inconsistency highlights the importance of considering contextual factors and demographic characteristics when interpreting study findings. The alignment of our results with those of previous studies conducted by Thapa *et al.* (2018) [10] and Basri *et al.* (2022) [11] indicates a degree of consistency in the factors influencing contraceptive knowledge among rural women. Factors such as education level, access to healthcare services, and the source of information play crucial roles in shaping women's understanding of contraception. Tailored interventions aimed at addressing knowledge gaps should take into account these factors to effectively empower women to make informed decisions about their reproductive health.

We found that the number of children a woman has did not exhibit a significant association with knowledge levels regarding contraception. This suggests that the mere count of children may not necessarily correlate with a woman's understanding of birth control methods. However, it's crucial to acknowledge that family planning decisions are multifaceted and may be influenced by factors beyond knowledge alone, such as cultural beliefs, access to healthcare services, and socio-economic status. Similarly, variables such as religion and socioeconomic status were not significantly associated with variations in contraceptive knowledge among the surveyed women in our study. While these factors may impact access to resources and opportunities, their direct influence on contraceptive knowledge may be mediated by other variables such as education level and access to healthcare. Additionally, the type of family structure, whether nuclear or joint, did not show a significant association with knowledge levels. This suggests that while family dynamics and communication patterns within different family structures may play a role in shaping individuals' access to information about reproductive health, the structure itself may not directly influence knowledge levels. Our findings align with those of a study conducted by Passah (2020) *et al.* [12], which also found no significant association between the number of children and contraceptive knowledge among rural women. This consistency suggests that factors beyond family size may be more influential in shaping contraceptive knowledge. Additionally, our results are consistent with the findings of other studies conducted by Singh *et al.* (2023) [13].

## Conclusion:

Overall, while certain demographic variables showed significant associations with contraceptive knowledge in our study, other factors may also contribute to individuals' understanding of birth control methods. Future research could explore the interplay between various socio-demographic factors and their collective influence on contraceptive knowledge among rural women, providing a more comprehensive understanding of the determinants of reproductive health outcomes in these communities.

## Figures and Tables

**Table 1:** Socio-demographic characteristics

**Socio-Demographic Variable**	**Components**	**Frequency**	**Percentage**
1.Age [years]	18-25	17	17%
	26-35	59	59%
	36-40	21	21%
	41-45	3	3%
2.Number of Children	No child	7	7%
	1	58	58%
	2	29	29%
	More than 2	6	6%
3.Religion	Hindu	100	100%
	Muslim	0	0%
	Christian	0	0%
	Other	0	0%
4.Occupation	Housewife	81	81%
	Self-employment	6	6%
	Govt. employment	7	7%
	Private employment	6	6%
5.Type of Family	Nuclear	43	43%
	Joint	57	57%
6.Source of Information	Mass media	23	23%
	Health personnel	13	13%
	Friends & family	64	64%
7.Socioeconomic Status	Upper class	8	8%
	Middle class	83	83%
	Lower class	9	9%
8.Education	Illiterate	3	3%
	Primary	19	19%
	High secondary	41	41%
	Graduate and above	37	37%

**Table 2:** Mean score and Mean percentage and the Standard deviation of Domain wise knowledge

**Topic**	**Maximam score**	**Mean score**	**Mean [%]**	**SD**
Introduction	5	4.7	83.40%	0.697
Mala N & Mala D	5	2.63	52.60%	1.186
Ezy Pills	4	2.45	63.50%	0.834
Saheli Pills	2	0.45	22.50%	0.626
Side effects	2	1.34	67%	0.67
Advantages/Disadvantages	2	0.84	42%	0.762
